# Online engagement and performance on formative assessments mediate the relationship between attendance and course performance

**DOI:** 10.1186/s41239-021-00307-5

**Published:** 2022-01-17

**Authors:** Chang Lu, Maria Cutumisu

**Affiliations:** Department of Educational Psychology, Centre for Research in Applied Measurement and Evaluation, Faculty of Education, University of Alberta, 6-102 Education Centre North, Edmonton, T6G 2G5 Canada

**Keywords:** Attendance and performance, Engagement, Formative assessment, Learning analytics, Learning management system

## Abstract

In traditional school-based learning, attendance was regarded as a proxy for engagement and key indicator for performance. However, few studies have explored the effect of in-class attendance in technology-enhanced courses that are increasingly provided by secondary institutions. This study collected n = 367 undergraduate students’ log files from Moodle and applied learning analytics methods to measure their lecture attendance, online learning activities, and performance on online formative assessments. A baseline and an alternative structural equation models were used to investigate whether online learning engagement and formative assessment mediated the relationship between lecture attendance and course academic outcomes. Results show that lecture attendance does not have a direct effect on academic outcomes, but it promotes performance by leveraging online learning engagement and formative assessment performance. Findings contribute to understanding the impact of in-class attendance on course academic performance and the interplay of in-class and online-learning engagement factors in the context of technology-enhanced courses. This study recommends using a variety of educational technologies to pave multiple pathways to academic success.

**Supplementary Information:**

The online version contains supplementary material available at 10.1186/s41239-021-00307-5.

## Introduction

Lecture attendance in higher education has been extensively researched in the past decades across various disciplines to develop attendance policy that maximizes instructional efficacy (e.g., Pérez-López & Ibarrondo-Dávila, 2020; Thatcher et al., 2007). The findings were mixed, with most previous studies showing a positive correlation between attendance and academic performance (Credé et al., 2010; Guleker & Keci, 2014), some showing minimal correlation (Choi-Lundberg et al., 2020), and some showing no correlation (van Walbeek, 2004) between attendance and academic achievement.

The pedagogical revolution from traditional face-to-face instruction to technology-enhanced learning (TEL) further complicates this issue, prompting researchers to re-evaluate the importance of in-class attendance and participation in the context of TEL-based courses, where more online learning factors are introduced. In traditional school-based courses, students must attend lectures to access educational resources, such as materials, peer interactions, and lectures. In contrast, the prevalent use of educational technologies makes resources available increasingly outside the classroom on different learning management systems (LMSs). Accordingly, more researchers hold that in-class attendance no longer matters for academic performance (Doggrell, 2020). Meanwhile, some researchers argue that, apart from the potential benefits, the availability of online learning resources including the asynchronous materials, online discussion forums, and formative assessments could induce students’ disengagement in school-based learning, and then cause failures in course achievements (Bergdahl et al., 2020). The debate on the relationship between attendance and academic performance in TEL-based courses has not been settled yet.

The COVID-19 pandemic makes online education ubiquitous especially in higher education and, concomitantly, in-class attendance untenable. Therefore, it is particularly important to understand how in-class lecture attendance affects academic performance and which TEL factors (e.g., access to asynchronous materials and formative assessments) may mediate this relationship. The present study sets out to examine the relationships among attendance, online learning engagement, online formative assessment performance, and course academic performance. More specifically, this study is guided by the following research questions:

1. Does in-class attendance predict academic performance?
2. Does LMS engagement mediate the relationship between attendance and academic performance?
3. Does performance on formative assessments mediate the relationship between attendance and academic performance?

## Theoretical framework

Among studies on technology-enhanced learning (TEL), engagement and motivation have been regarded as two essential components (Bedenlier et al., 2020; Lan & Hew, 2020). Several theories were employed to address the relationship between motivation and learning, such as expectancy-value theory (Eccles & Wigfield, 2002; Wigfield & Eccles, 2000), goal-orientation theory (Dweck, 1986, 1989, 1992), and self-determination theory (Deci & Ryan, 1985, 2004, 2012). Expectancy-value theory defines motivation by two main factors: expectancy, which refers to individuals’ expectations of success (i.e., the degree to which they believe they will be successful) and value, which refers to individuals’ perceived value or interests in completing tasks (Cook & Artino Jr, 2016). Goal-orientation theory is a socio-cognitive theory of motivation that explicates motivation via three types of goals: mastery-oriented goals (i.e., how to master skills), performance-oriented goals (i.e., how to perform better than others and receive positive judgments), and performance-avoidance goals (i.e., how to avoid failure; Cook & Artino Jr, 2016). Self-determination theory (SDT) is another motivation theory that assumes outcomes are influenced by three universal and basic human needs: autonomy, competency, and relatedness (Deci & Ryan, 1985).

The theory of engagement and motivation is currently absent for TEL (Hew et al., 2019). Thus, previous studies on TEL-based learning tried to explain findings through the theories discussed above. For example, Berweger et al. (2021) adopted expectancy-value theory to examine the relationship between 95 university students’ specific expectancy-value appraisals and achievement emotions. They found that high expectations for success and high interest in tasks were directly associated with positive emotions and inversely associated with negative emotions. Drawing on self-regulated learning and goal-orientation theories, Lin (2021) evaluated 558 Taiwanese university students’ online learning tasks, value, goal orientation, and self-efficacy before and after the COVID-19 outbreak. The author developed and validated an instrument named “COVID-19 Online Learning Motivation (COLM)” questionnaire to measure online learning task value, goal orientation, and self-efficacy through ten subscales including *Attainment value* subscale, *Utility value* subscale, *Intrinsic value* subscale, and *Mastery-approach goal* subscale, etc. Findings revealed that students showed increased endorsement with *Attainment value*, *Utility value*, *Mastery-approach goal*, *Mastery-avoidance goal*, *Performance-avoidance goal*, and *Functional self-efficacy* during the transitions from traditional classroom-based learning to online learning after the COVID-19 outbreak (Lin, 2021).

Regarding student engagement and motivation in online learning environments and TEL-based learning, most of the previous studies adopted SDT to explain their findings (Chen & Jang, 2010; Chiu, 2021a, 2021b; Reeve, 2012; Sun et al., 2019). Thus, we grounded our research in SDT. The following sections provide an in-depth discussion of SDT and previous investigations on SDT and learning outcomes, with a focus on TEL-based learning.

### Definition and core concepts of SDT

Self-determination Theory (SDT) is a motivation theory proposed by Deci and Ryan (1985, 2004, 2012). SDT conceptualizes three universal and basic needs of humans for outcomes: autonomy, competency, and relatedness (Deci & Ryan, 1985). Autonomy refers to a sense of control and agency. Competency shows that one is feeling confident to complete a task. Relatedness is the feeling of included and connected with others. SDT hypothesized that students will be more self-determined when their psychological needs are satisfied, and therefore will be more likely to be motivated and yield positive outcomes among various contexts. In contrast with other motivation theories, SDT treats motivation as a continuum that falls into three categories: intrinsic motivation, extrinsic motivation, and amotivation. Intrinsic motivation is the state of performing tasks out of enjoyment, satisfaction, and self-fulfillment (i.e., self-determined), extrinsic motivation refers to the state of performing tasks out of compliance, external rewards, or punishment (i.e., self-control), and amotivation is the state of lacking motivation (i.e., non-self-determined).

### SDT and learning outcome

According to SDT, more autonomous forms of motivation will lead to higher levels of students’ engagement and learning across various educational and cultural contexts (Ryan & Deci, 2020). Previous studies mainly tested the SDT among traditional school contexts and concluded that students engage more in learning activities and achieve higher performance if the pedagogical practices adequately address the basic psychological needs of students and promote autonomous motivation (Cerasoli & Ford, 2014; Cerasoli et al., 2014; Jang et al., 2012; Reeve, 2002, 2013; Vasconcellos et al., 2020). Compared with studies situated in traditional school-based contexts, few studies explored SDT and its applications in the technology-enhanced learning (Chiu, 2021a; Ryan & Deci, 2020). Chen and Jang (2010) used structural equation modeling to examine Deci and Ryan’s SDT model among n = 262 learners from two online teaching certificate programs. Findings of their study confirmed the dynamics among contextual support, psychological needs, motivation, and learning outcomes theorized by SDT. More specifically, the effect of need satisfaction mediates the relationship between contextual support and motivation (i.e., self-determination). However, the self-determined motivation failed to predict learning outcome. On the other hand, Hsu et al. (2019) modified Chen and Jang’s model by adopting alternative conceptualizations and operationalization of the key variables and re-examined the impact of SDT among n = 330 undergraduate students’ academic performance. Results of their study supported that enhanced self-determination motivation facilitated the satisfaction of the SDT basic psychological needs and higher levels of student engagement, which were positively associated with higher perceived knowledge transfer and better course performance. Ryan and Deci (2020) highlighted the promises and importance to study SDT in technology-enhanced education. The main challenges for future SDT research include how to retain students’ attention and creating more engagement for learning tasks to enhance motivation through various learning technologies.

As a key component of SDT, engagement refers to individuals’ levels of endeavor and involvement in their own learning (Fredricks et al., 2004; Wang et al., 2017). Engagement can be categorized into four dimensions: emotional, behavioral, cognitive, and social dimensions (Fredricks et al., 2016; Wang et al., 2016, 2019). The emotional dimension refers to individuals’ positive or negative mental states when confronted with peers, teachers, learning, and feedback. The cognitive dimension includes individuals’ cognitive skills exerted on learning including thinking, applying, connecting, understanding, and reflecting. The behavioral dimension refers to taking actions such as participating in class, concentrating, and making efforts to learn (Fredricks et al., 2016). Lastly, the social dimension (Wang et al., 2016) focuses on the interaction or collaboration with peers.

Student engagement is often operationalized by indicators such as school attendance, activity participation, and social interactions in traditional schools (Wang et al., 2016), whereas TEL often conceptualizes engagement as time invested on learning environments (Henrie et al., 2018), online interactions with different modules, peers, or teachers (Hung & Crooks, 2009; Pellas, 2014), postings and discussions on the forums (Broadbent & Poon, 2015), or self-assessment (Kibble, 2007; Zacharis, 2015). Empirical evidence shows that active student engagement substantially promotes academic performance (Fredricks et al., 2004, 2016; Wang et al., 2017). In contrast, disengagement is defined by absenteeism, withdrawal, school dropout, and low interactions with the online learning environments, which are often associated with low academic performance (Skinner et al., 2009; Wang et al., 2016).

The present study probes the impact of students’ in-class engagement (i.e., class attendance as proxy) on course performance. Further, this study examines whether and how educational technology potentially mediates the effect of traditional in-class engagement on academic performance. The rest of the paper is organized as follows. First, empirical studies are reviewed on the impact of class attendance and online engagement on academic performance, which align with our theoretical framework. Other confounding predictor for performance is also discussed. Second, the research questions and hypotheses are outlined, followed by the proposed learning analytical methods. Then, the results and discussion are presented. Lastly, the conclusion, educational implication, and recommendations are provided.

## Literature review

### Attendance and performance

The relationship between attendance and academic performance in higher education has been explored extensively for decades. The debate on whether it is necessary to require mandatory attendance in secondary institutions has been going on concurrently. Most previous studies found a positive relationship between attendance and academic performance (Devadoss & Foltz, 1996; Kirby & McElroy, 2003). Romer (1993) first found a significant positive correlation between performance and attendance based on an analysis of n = 195 undergraduate students’ attendance and course performance and advocated for mandatory attendance in school to promote performance. Durden and Ellis (1995) then defined attendance as a proxy for motivation. They collected n = 346 students’ self-reported absence records, examined the relationship between attendance and academic achievement in an economics course, and found that absenteeism led to poor academic performance. Credé et al. (2010) later conducted a systematic review on 69 empirical studies and found that lecture attendance was a significant medium-strong predictor of academic performance, before and after controlling for other potential confounding variables, such as student age, gender, grade, SAT score, IQ, hours of employment, and motivation levels. More recently, similar findings were also presented by studies across different subjects with varying effect sizes (Hollett et al., 2020; Louis et al., 2016). For example, Landin and Pérez (2015) recruited four cohorts of university students from a pharmacy course and correlated their attendance with performance separately. Positive correlations were observed across all four cohorts, suggesting a positive effect of attendance on performance.

Andrietti (2014) also analyzed longitudinal data from undergraduate students enrolled in an introductory macroeconomic course across the academic year to evaluate the relationship between lecture attendance and academic performance using proxy variable regressions. Findings revealed that attendance had a moderate positive impact on performance, although the effect disappeared after introducing time-invariant variables. This suggests that unobservable mechanisms such as students’ characteristics or motivation may interact with the relationship between attendance and performance. Similarly, Krohn and O’Connor (2005) observed students in three undergraduate macroeconomics courses and found a positive significant effect of attendance. However, the relationship became non-significant when instrumental variable techniques were applied to analyze the data collected during the term.

No relationship as well as minimal or conditional relationships between attendance and performance have increasingly been found in recent studies (Andrietti & Velasco, 2015; Büchele, 2020; Choi-Lundberg et al., 2020; van Walbeek, 2004) and were attributed to two main reasons. First, with the world-wide digitalization of education, students no longer must attend classes to gain access to course materials, so attendance is not vital for achievement (Büchele, 2020). Second, unlike previous studies that only correlate performance with attendance, more studies seek to address the endogenous bias of attendance by controlling confounding variables, such as student characteristics and motivations (Choi-Lundberg et al., 2020), or introducing mediators that are related to engagement, such as task engagement, tutorial engagement, and metacognition regulation (Büchele, 2020).

On the other hand, Schneider and Preckel (2017) argued that the effect of attendance on learning outcomes has remained significant and withstood the great advance of learning technologies over the years. They conducted a systematic review of 38 meta-analyses to investigate the variables associated with achievement in higher education. Class attendance (*d* = .98, ranked 6) ranked the sixth most significant predictor for academic achievement among all the 105 variables examined, and ranked the most significant predictor within student variable category. In addition, their study revealed that online courses and blended courses does not seem to mitigate the importance of class attendance for academic achievement. However, they argued that it is still too early to draw conclusions on mandatory attendance policies before the mechanism underlying class attendance has been fully understood when information and educational technology overtake the field of education.

Undoubtedly, attendance has been proved to impact performance. However, there are still some unresolved issues that remain to be further studied on this topic. First, most previous studies adopted self-reported attendance records as predictors of academic achievement, in which researchers requested participants to recall their attendance rate at the end of the semester. The self-reported attendance rate introduces measurement bias. Second, attendance is an endogenous factor for learning, with highly motivated and high-achieving students being more likely to attend lectures regularly and engage in the class contents, and thus, achieve higher course performance (Andrietti, 2014). Although some studies attempted to control student-level variables to mitigate the upward endogeneity error of attendance, few incorporated the instruction-level variables, such as in-class activity engagement, peer or teacher interaction, or performance on formative assessments. The potential measurement error and endogeneity bias may severely attenuate the validity of the conclusions presented in the related research.

Based on the TEL engagement, in-class attendance serves as an indicator of traditional school engagement. Concomitantly, online engagement is indicated by self-regulated online learning activities and performance on online formative assessments. Both traditional and online engagement may be essential determinants of academic success. In the era of TEL-based education, more research needs to be done to understand the dynamics among in-class engagement, online engagement, and academic performance. Moreover, the potential mediating effects of TEL engagement indicators upon the relationship between in-class engagement and academic performance are also underexplored.

### Self-regulated learning, formative assessment, and performance in TEL

Technology-enhanced learning has become a major trend in education, especially in today’s climate of the COVID-10 pandemic. TEL transforms the conditions of engagement learning from traditional classroom-based to blended and, currently, to fully online through various digital technologies (López-Pérez et al., 2011; Nouri et al., 2016). A substantial body of literature has investigated the relationships between student online self-regulated learning and self-assessment with academic performance using data extracted from LMSs (e.g., Hung & Crooks, 2009; Shi et al., 2015). Most previous studies have concluded consistent results with traditional schooling contexts that higher levels of TEL engagement could facilitate academic success (Hung & Crooks, 2009; Kibble, 2007; Zacharis, 2015). However, few have investigated the associations among traditional engagement indicators such as attendance, TEL engagement indicators such as self-regulated learning and self-assessment, and academic performance.

As an essential TEL engagement indicator, students’ online self-regulated learning plays an increasingly important role in the formal contexts of higher education, for LMSs such as Canvas, D2LBrightspace, Moodle, and Sakai have been regarded as critical digital tools that assists faculty members in delivering poly-synchronous materials, lectures, and assessments (Gautreau, 2011; Washington, 2019). Some of the studies have been done to evaluate the relationship between attendance, online learning engagement, and performance in online learning in higher education (Bekkering & Ward, 2020; Doggrell, 2020; Nieuwoudt, 2020). Doggrell (2020) inspected the associations between lecture attendance, lecture recordings access, and academic achievements on n = 117 medical students sampled from two sessions of medical laboratory science courses. They found that, with the availability of lecture recording, there is no significant correlation between lecture attendance and academic achievement. They suggested that using a mixture of multimedia educational technologies is likely to ensure higher academic success.

Online formative assessment is another important indicator of TEL engagement that predicts performance (Gikandi et al., 2011; Spector et al., 2016). Educators need to consider formative practices and optimally integrate them into their teaching and assessments. Online formative assessment also provides learners with self-evaluation and feedback to help them orient and adapt their own self-regulated learning (Zimmerman, 2002). Gikandi et al. (2011) conducted a review of literature on 19 empirical studies about online formative assessment in the context of online learning in higher education. They found that online formative assessment effectively promoted learner engagement and learner community development. Other studies also confirm the constructive, beneficial effect of formative assessment on learning outcomes (Rakoczy et al., 2019; Robinson & Udall, 2006).

With the fast development of the areas of educational data mining (EDM) and learning analytics (LA), a great number of studies emerged using EDM and LA to measure online engagement and learning by analyzing web-based log event data generated during the LMS usage recording the users’ activities, IP address, date, and time sequence (Aldowah et al., 2019; Dutt et al., 2017; Papamitsiou & Economides, 2014; Romero & Ventura, 2010; Romero et al., 2008). Common practices of EDM and LA include applying feature-engineering techniques to extract engagement indicators, such as analyzing the text posted on online forums (Larsen et al., 2008) or counting individuals’ click frequencies and total time spent in different LMS sessions throughout a course (Geigle & Zhai, 2017; Zacharis, 2015). Students’ online learning engagement can be objectively reflected by their actual web usage on the LMS. Most studies conducted using EDM/LA approaches reported that higher levels of self-regulated learning are positively correlated with academic performance (Geigle & Zhai, 2017; Hung & Crooks, 2009; Zacharis, 2015). However, few studies have explored the impact of online learning engagement as captured by features extracted from log data on the relationship between in-class lecture attendance and academic performance.

### Prior knowledge and performance

Prior knowledge is constantly regarded as a significant student characteristic to predict performance in TEL education (Asarta & Schmidt, 2017; Kinsella et al., 2017; Schneider & Preckel, 2017; Song et al., 2016; Spires & Donley, 1998; Tobias, 1994). Prior knowledge is defined as the information or experiences that a learner already established regarding a new topic either taught from learning or drawn from experiences (Tobias, 1994).

Previous studies commonly found that prior knowledge is positively related with academic performance through the facilitation of higher levels of motivation, engagement, and self-regulation (e.g., Schneider & Preckel, 2017; Song et al., 2016). Song et al. (2016) conducted a study to examine the effects of prior knowledge, self-regulation, and motivation on performance via structural equation modeling. They assessed 386 medical clerk students’ prior knowledge through multiple choice items and measured their self-reported self-regulation and motivation. A knowledge post-test and a clinical reasoning test were administered as performance measures. Findings revealed both direct and indirect positive correlations of prior knowledge with learning outcome and self-efficacy. Conversely, students with little or no prior knowledge will be disadvantaged when they process and memorize entirely new information. In the worst case, students with false prior knowledge will have to correct and update the false information and reconstruct their knowledge system (Kowalski & Taylor, 2009). From a systematic review of meta-analyses of variables associated with achievement in higher education, Schneider and Preckel (2017) also found that prior intelligence or prior knowledge is an important predictor for achievement (d = .90, ranked 7 out of 105).

Given the fact that prior knowledge is reported to account for a large proportion of variances of learning outcomes (Schneider & Preckel, 2017; Song et al., 2016; Tobias, 1994), the present study controlled the effect of prior knowledge when examining the relationship among attendance, self-regulated learning, performance of formative assessments, and academic performance to exclude the confounding bias.

### Gaps identified in the previous studies

With the rapid digitalization of education around the world, online learning and formative assessment have become essential components of both formal and informal learning in higher education and the key to academic success. The findings on impact of attendance on performance are no longer valid if online learning and online formative assessment are not considered and evaluated.

Moreover, most previous studies adopted an instrumental approach, such as the National Survey of Student Engagement (NSSE: Ewell, 2010; Kuh, 2009), the Australian Survey of Student Engagement (AUSSE: Coates, 2010), or the Utrecht Work Engagement Scale for Students (UWES-S: Carmona-Halty et al., 2019; Seppälä et al., 2009) to measure engagement and other student characteristics. The self-reported scales exhibit inherent measurement errors and may not reflect students’ real level of engagement.

The advancement of educational data mining (EDM) and learning analytics (LA) methods could provide more insights in TEL contexts. The LMS can be used to record attendance more accurately, compared with the self-reported attendance rate recalled by the students. In addition, researchers can extract students’ online learning activities from the automated generated log files in the LMS. The use of LMS also enables instructors to examine the students’ prior knowledge, to organize in-class online activities, to administer online formative assessments in and outside the classroom, and to revise their instruction because of the way students interact with the materials. The collection of all the information above through LMS and its inclusion into the analysis of attendance and performance can help minimize the endogeneity and measurement bias mentioned in previous studies.

Thus, we propose a learning analytics approach to measure students’ lecture attendance and online learning engagement through information extracted from the log file generated by LMS. Additionally, we regard TEL engagement indicators—self-regulated online learning and online formative assessment administered on LMS—as important indicators of academic performance in addition to traditional engagement indicator attendance.

### The present study

We propose a novel method that uses a learning analytic approach to mine the LMS log data to extract event-based variables that record students’ in-class attendance, measure their online learning engagement, and collect their performance on online formative assessments. With the measures extracted from the LMS log data, we investigate the associations among attendance, online learning engagement, performance on formative assessment, and course academic performance. This study makes the following hypotheses:

- Hypothesis 1: In-class attendance positively predicts the final course score.
- Hypothesis 2: LMS engagement positively mediates the relationship between in-class attendance and the final course score.
- Hypothesis 3: Performance on formative assessments positively mediates the relationship between in-class attendance and the final course score.

## Methods

### Participants and procedure

Participants were n = 367 Elementary and Secondary Education undergraduate students at a large university from Western Canada enrolled in three sections of an undergraduate mandatory Educational Assessment course in Winter 2019. Participants were recruited using convenience sampling, as one of the authors was the instructor of the course. Students were required to bring a digital device to class to participate in the interactive classroom activities, keep up with class readings, complete tasks, and participate in class discussions. Class attendance was not mandatory, but highly encouraged to engage participants in classroom discussions, involve them in class activities to facilitate their understanding of complex concepts and intricacies of classroom assessment. There were 13 lectures during the term, and each lecture started with a formative quiz administered on Moodle. On the first day of class, a prior knowledge quiz was administered on Moodle. Then, each lecture started with a quiz testing the material taught in the previous lecture on Moodle. Each quiz was timed, and it was opened at the beginning of each lecture. Students who physically attended were instructed to complete the in-class quiz at the beginning of each class. Attendance was operationalized as the timestamp of the start of the quiz on Moodle. Asynchronous course materials including the syllabus, assignments, lecture notes, handouts, external links, formative quizzes, and a discussion forum were available on Moodle. The instructor also provided weekly in-class lectures interspersed with hands-on individual and group activities.

### Data sources

Log data of the three course sections was downloaded by a third party and anonymized before the analyses commenced. After pre-processing the log file, the dataset included the following variables: Student ID (i.e., corresponding to each student), Activity Name (i.e., corresponding to different Moodle modules), Activity Context (i.e., corresponding to specific actions within the Moodle), IP address, and Timestamp in the format of Year-Month-Date and time. The study was approved by the University of Alberta’s Human Research Ethics Board (Pro00095249).

### Measures

#### Attendance

Students’ lecture attendance was measured by the timestamp of the quiz attempt at the beginning of each lecture. More specifically, students were counted as absent if their IP address did not match the IP of the classroom; otherwise, they were counted as present.

#### Prior knowledge

Students’ knowledge of the course material was measured using the scores on the Prior Knowledge quiz administered in the first day of the course, at the beginning of the first lecture*.* The prior quiz consisted of 11 questions, with each question testing the material of a corresponding lecture during the term.

#### Online learning engagement

The latent variable students’ levels of engagement in the LMS were measured by total click frequencies of different LMS modules accessed, including *File* (i.e., accessing, viewing, and downloading lecture notes and other course materials), *Forum* (i.e., posting or viewing the content of the discussion forum), *URL* (i.e., clicking on an external link posted on the Moodle), and *Assignment* (i.e., accessing, viewing, or submitting an assignment or viewing feedback on an assignment).

#### Formative assessment performance

Students’ online quiz scores were calculated for every lecture. A total of twelve formative quizzes were administered in class, in addition to the Prior Knowledge Quiz. The quizzes were reopened by the instructor after each lecture, so that students could access and review the formative assessments in preparation for the midterm and final exams. The online formative quizzes were only designed for students to practice the material learned in class and they did not count towards their final grade.

#### Course academic performance

Students’ final scores in the class were collected, ranging from 0 to 100 and included two assignment scores, a midterm exam score, and a final exam score.

### Data analysis

Data normalizations were performed prior to statistical analyses because the scales of the variables included in the study varied significantly (e.g., the click frequencies of behavior-based variables, attendance frequencies, and the performance measures). All selected variables were normalized to the scale of 0–1.

Then, two Structural Equation Model (SEM) were fitted to the dataset. The Baseline Model (M0) or null model testifies the direct effect of *Attendance* on *Academic Performance*, whereas all the other structural path coefficients from were fixed to zeros. The Mediation Model (M1) examined the mediating effect of *Online Learning Engagement* and *Formative Assessment Performance* on the relationship between *Attendance*, and *Course Academic Performance*. The measurement model examined the factor loadings of online learning activities extracted from different LMS modules on the latent factor *Online Learning Engagement*. The structural model examined whether: (1) *Attendance* directly predicted *Course Academic Performance*; and (2) *Learning Engagement* and *Formative Assessment Performance* mediated the relationship between *Attendance* and *Course Academic Performance*. For both SEMs, we controlled for the *Prior Knowledge* covariate in the model.

The SEM analysis was conducted using the *sem* function within the *lavaan* package (Rosseel, 2012) in *R*. The model was estimated using the Robust Maximum Likelihood. The model fitness was evaluated by the Chi-Square fit index test, Goodness of Fit Index (GFI), Adjusted Goodness of Fit Index (AGFI), Comparative Fit Index (CFI), Tucker Lewis index (TLI), Root Mean Square Error of Approximation (RMSEA), and Root Mean Square Residual (RMSR; Hu & Bentler, 1998, 1999). Model comparisons between the two SEMs were performed using Akaike Information Criterion (AIC; Akaike, 1974) and Bayesian Information Criterion (BIC; Schwarz, 1978).

## Results

### Summary of descriptive statistics

The top of Table 1 presents the descriptive statistics of participants’ raw course attendance frequency and click frequencies of different LMS modules. Out of the 13 lectures in the Winter 2019 term, participants attended 10.57 lectures on average with an *SD* of 3.09. Among all the modules, participants were most active in accessing, viewing, or downloading the course materials including lecture notes, handouts, and reading materials (*Mean* = 84.64, *SD* = 40.17), followed by activities related to the assignments (*Mean* = 27.92, *SD* = 10.59), extra curriculum URLs (*Mean* = 16.17, *SD* = 12.22), and lastly, discussion forum (*Mean* = 3.22, *SD* = 6.61).

**Table 1:** Descriptive statistics of the observed variables

Week		*Mean*	*SD*
1–13	Attendance	10.57	3.09
1–13	LMS module: file	84.64	40.17
1–13	LMS module: assignment	27.92	10.59
1–13	LMS module: forum	3.22	6.61
1–13	LMS module: URL	16.17	12.22
1	Prior knowledge quiz	48	22
2	Formative quiz: review of lecture 1	82.43	18.36
3	Formative quiz: review of lecture 2	80.99	19.15
4	Formative quiz: review of lecture 3	65.89	25
5	Formative quiz: review of lecture 4	78.96	20.99
6	Formative quiz: review of lecture 5	81.3	24.56
7	Formative quiz: review of lecture 6	84.62	24.49
8	Formative quiz: review of lecture 7	70.42	22.69
9	Formative quiz: review of lecture 8	82.5	23.84
10	Formative quiz: review of lecture 9	60	28.89
11	Formative quiz: review of lecture 10	76.68	23.43
12	Formative quiz: review of lecture 11	75.47	25.19
13	Formative quiz: review of lecture 12	74.89	26.23
2–13	Quiz (average score of the 12 formative quizzes)	73.99	24.56
15–17	Course final score	82.02	7.73

A summary of the prior knowledge quiz, the twelve formative quizzes, and the course final scores are shown at the bottom of Table 1. The prior knowledge quiz, the 12 formative quizzes, and the course final score were measured on a scale of 0–100 before variable normalization. The prior knowledge quiz had the lowest average score, whereas the average scores of the 12 quizzes ranged from *Mean* = 60 (*SD* = 28.89) to *Mean* = 84.62 (*SD* = 24.49). In general, there were large individual variations on the formative assessment scores. Participants’ course final score had a mean of 82.02 and a *SD* of 7.73 (Table 2).

**Table 2:** Bivariate correlations among the observed variables

	Attendance	Quiz	File	Forum	URL	Assignment
Quiz	0.27***					
File	0.21***	0.05				
Forum	0.19***	0.11*	0.17***			
URL	0.29****	0.18***	0.44***	0.21***		
Assignment	0.07	0.03	0.33***	0.07	0.26***	
Course final score	0.21****	0.50***	0.11*	0.12*	0.31***	0.14**

*Quiz* Formative assessment performance

****p < .0001, ***p < .001, **p < 01, *p < 0.5, two-tailed Pearson correlation

Table 3 presents the bivariate Pearson correlations among the observed variables. Results show that the observed variables *File*, *Forum*, *URL*, and *Assignment* underlying the latent construct *Online Learning Engagement* are significantly correlated with each other with small to medium effect sizes, which suggests that the observed LMS variables can load on the single latent variable without multicollinearity. In addition, participants’ *Formative Assessment Performance* is only significantly correlated with URL (*r* = .18, *p* < .001), but not significantly correlated with other LMS variables including *File* (*r* = .05, *p* > .05), *Forum* (*r* = .10, *p* > .05), and *Assignment* (*r* = .03, *p* > .05), indicating that the two variables *Online Learning Engagement* and *Formative Assessment Performance* represent two distinct constructs relevant to TEL engagement. The in-class engagement indicator *Attendance* is significantly correlated with the outcome variable *Course Academic Performance* (*r* = 0.21, *p* < .001) and all the TEL engagement indicators except for *Assignment* (*r* = .007, *p* > .05). Thus, there are potential interactions among class attendance, online engagement, performance on formative assessment, and course final score. Lastly, the outcome variable *Course Academic Performance* has significant positive correlations with all the selected predicting variables, which shows that both in-class engagement indicator and online learning/TEL indicators could have positive impacts on academic performance. The results from the Pearson correlations lay the foundations for the following SEM analyses.

**Table 3:** SEM fit index summary

Model	χ^2^	*df*	GFI	AGFI	CFI	TLI	SRMR	RMSEA	AIC	BIC
M0	207.69	21	0.85	0.74	0.47	0.31	0.14	0.16	− 3113.52	− 3066.66
M1	35.30	17	0.97	0.93	0.95	0.92	0.06	0.05	− 3277.91	− 3215.42

*M0* baseline model; *M1* the mediation model

### Summary of SEM results

The model fit indices and the error terms of the two SEMs are presented in Table 3. When RMSEA < .08, GFI, AGFI, CFI and TLI > .90, and SRMR < .08, the model is regarded as fitting well (Marsh et al., 1988). Results show that the Mediation Model (M1: ${X}^{2}$ = 35.30, *df* = 17, GFI = 0.97, AGFI = 0.93, CFI = 0.95, TLI = 0.92, RMSEA = 0.05, and SRMR = 0.06) yielded good model fit, whereas the Baseline Model (M0: ${X}^{2}$ = 207.69, *df* = 21, GFI = 0.85, AGFI = 0.74, CFI = 0.47, TLI = 0.31, RMSEA = 0.16, and SRMR = 0.14) fitted poorly to the data. To further compare the relative fit indices, AIC and BIC were computed. Both AIC and BIC are relative indices that are penalized by the number of parameters. The smaller AIC and BIC values are, the better the model fits the data. Table 3 shows that the Mediation model (AIC = − 3113.52; BIC = − 3066.66) outperformed the Baseline model (AIC = − 3277.91; BIC = − 3215.42) with lower AIC and BIC. To conclude, the Mediation model better fits the data than the Baseline model does. The following section summarizes the measurement and structural models of the two SEMs and answers the research questions based on the three hypotheses.

#### Hypothesis 1

In-class attendance positively correlates with the final course score.

**Fig. 1:**
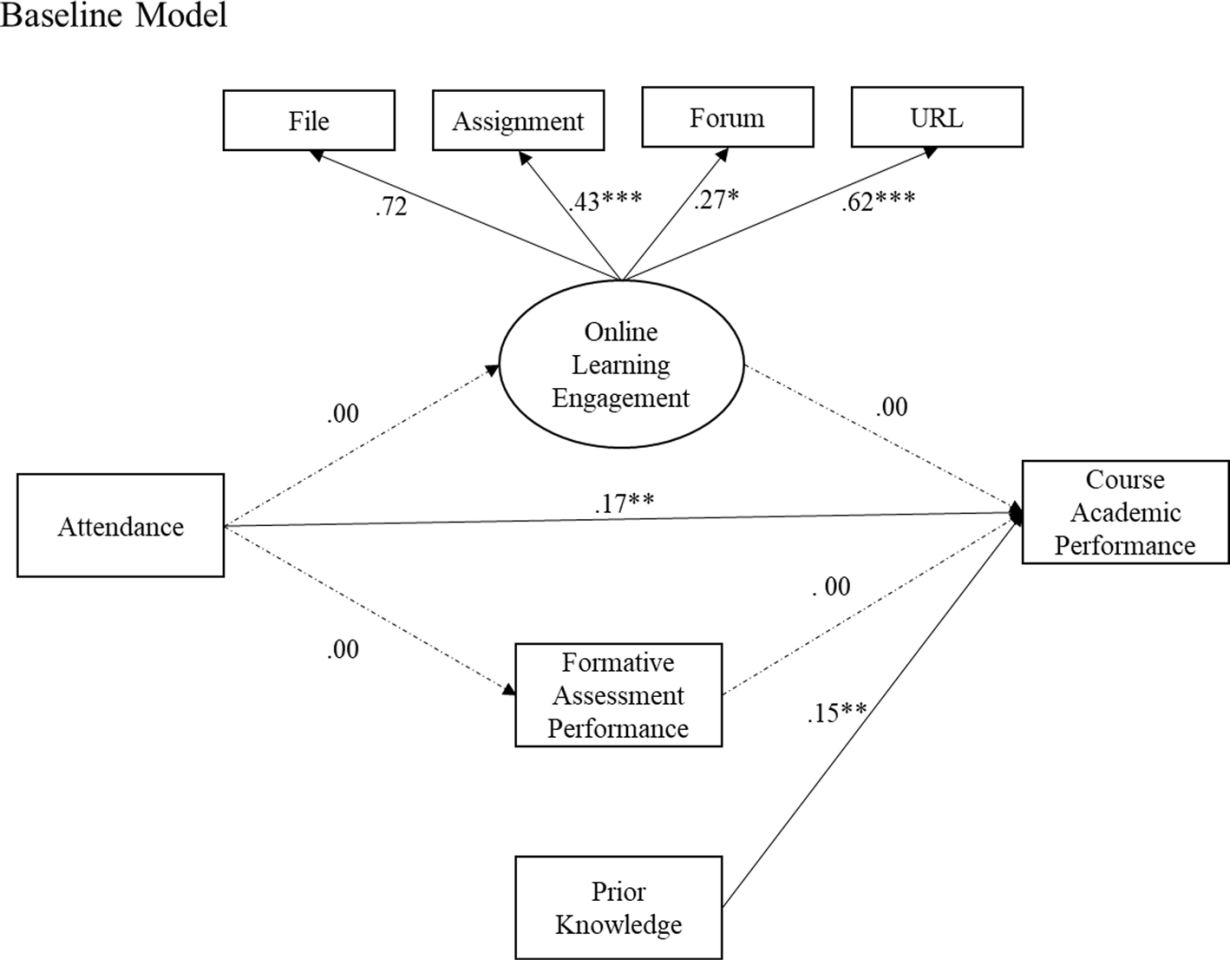
SEM path diagram of baseline model

Table 4 presents the summary of the Baseline model, in which the structural coefficients between *Online Learning Engagement*/*Formative Assessment Performance* and *Course Final Score* were fixed to zeros. Both unstandardized and standardized model coefficients were reported. Results show that in-class *Attendance* (β = 0.17, *p* < .01) and *Prior Knowledge* (β = 0.15, *p* < .01) are positively correlated with the *Course Academic Performance*, when excluding the effects of *Online Learning Engagement* and *Formative Assessment Performance*. The two predictors prior knowledge and in-class attendance account for 6% variance of the outcome variable *Course Final Score* (${R}^{2}$ = 0.06, *p* < .001). Therefore, Hypothesis 1 is confirmed that in-class attendance positively correlates with the final course score, when TEL indicators are excluded. The path diagram of the Baseline model is plotted in Fig. 1 in standardized coefficients.

**Table 4:** SEM: the baseline model

Measurement model						
Latent variable	Observed variable	b	***SE***	β	***p***	${R}^{2}$
Online learning engagement						
	File	1.00		0.72		
	Forum	0.29	0.12	0.27	< .05	
	URL	1.39	0.27	0.62	< .001	
	Assignment	0.69	0.14	0.43	< .001	

Structural model						
Dependent variable	Independent variable	b	***SE***	β	***p***	${R}^{2}$
Online learning engagement						
	Attendance	0.00	–	0.00	–	
Formative assessments performance						
	Attendance	0.00	–	0.00	–	
Course final score						0.06***
	Attendance	0.11	0.04	0.17	< .01	
	Online learning engagement	0.00	–	0.00	–	
	Formative assessments performance	0.00	–	0.00	–	
	Prior	0.11	0.04	0.15	< .01	

***p < .001

LMS engagement and performance on formative assessments positively mediates the relationship between in-class attendance and the final course score.

**Fig. 2:**
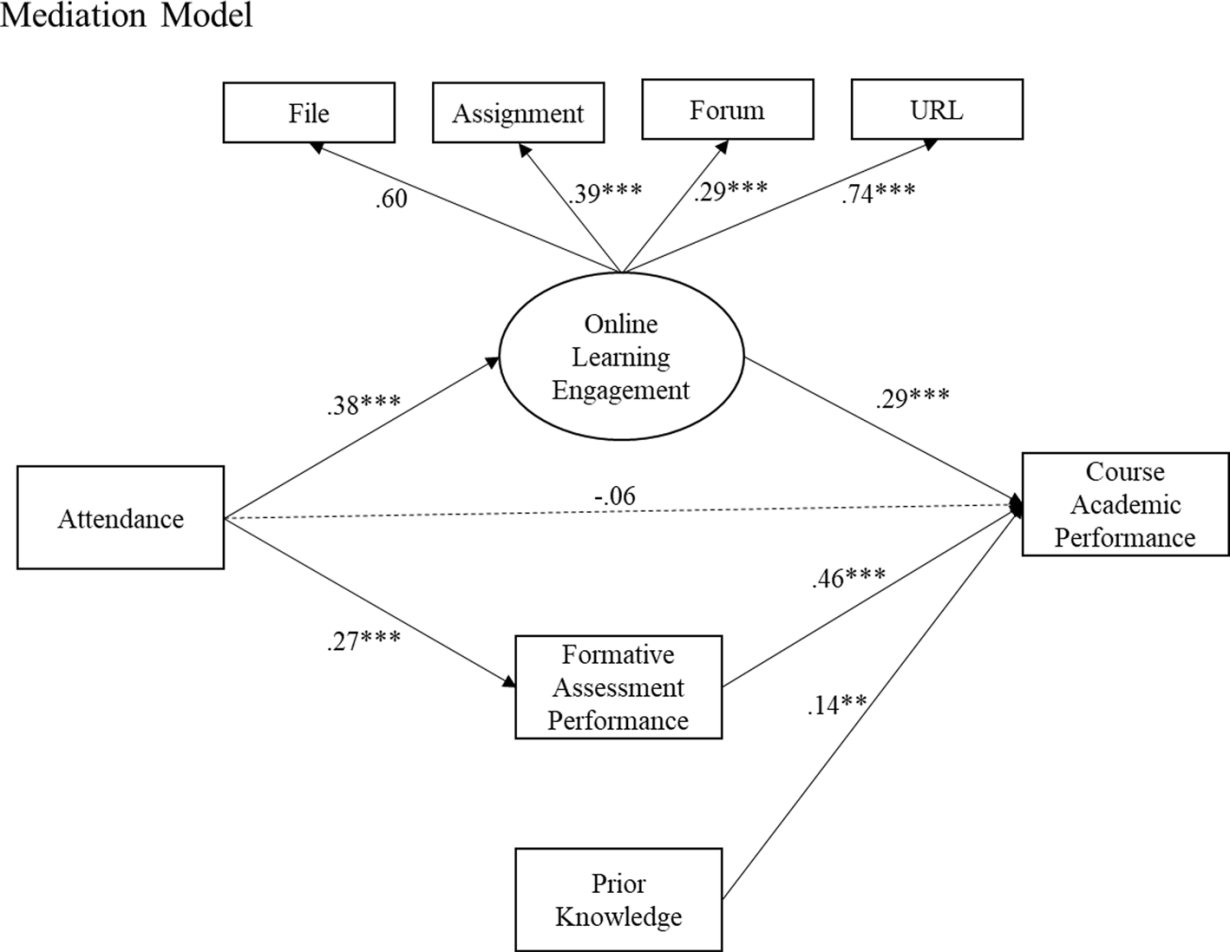
SEM path diagram of the mediation model

The Mediation model addresses the mediating effect of LMS engagement and formative assessment performance on the relationship between in-class attendance and final course score. Compared with the Baseline model, the total variance explained of *Course Final Score* by the Mediation model increased to 32% (${R}^{2}$ = 0.32, Δ${R}^{2}$ = 0.26, *p* < .001). Table 5 presents that after controlling for the effects of *Prior Knowledge*, *Online Learning Engagement*, and *Formative Assessment Performance*, in-class attendance has no significant direct impact on *Course Final Score* (β = − 0.06, *p* = .437) in the Mediation model. The non-significant path coefficient indicates that the effect of in-class attendance on final course score is fully mediated by *Online Learning Engagement* and *Formative Assessment Performance*. More specifically, the structural model shows that 14% of the variance of *Online Learning Engagement* (${R}^{2}$ = 0.14, *p* < .001) can be explained by the *Attendance*, whereas 7% of the variance of *Formative Assessment Performance* (${R}^{2}$ = 0.07, *p* < .001) can be explained by *Attendance*. In addition, the structural coefficients revealed that the indirect effect of *Attendance* on *Course Final Course* is mediated by both *Online Learning Engagement* (β = (.38)*(.29), *p* < .001) and *Performance on Formative Assessments* (β = (.27)*(.46), *p* < .001). Thus, both Hypotheses 2 & 3 were confirmed. The controlled variable *Prior Knowledge* also positively predicted *Course Final Score* (β = 0.14, *p* < .01). Figure 2 plotted the standardized coefficients of the Mediation model.

**Table 5:** SEM: the mediation model

Measurement model						
Latent variable	Observed variable	b	***SE***	β	***p***	${R}^{2}$
Online learning engagement						
	File	1.00		0.60		
	Forum	0.37	0.13	0.29	< .001	
	URL	1.97	0.34	0.74	< .001	
	Assignment	0.74	0.15	0.39	< .001	

Structural model						
Dependent variable	Independent variable	b	***SE***	β	***p***	${R}^{2}$
Online learning engagement						0.14***
	Attendance	0.10	0.02	0.38	< .001	
Formative assessments performance						0.07***
	Attendance	0.15	0.04	0.27	< .001	
Course final score						0.32***
	Attendance	− 0.04	0.05	− 0.06	.437	
	Online learning engagement	0.71	0.34	0.29	< .001	
	Formative assessments performance	0.55	0.07	0.46	< .001	
	Prior	0.10	0.03	0.14	.003	

***p < .001, **p < 01, *p < 0.5

In terms of the measurement model, the top of Table 5 revealed that all selected LMS modular variables contributed significantly to the variation of the latent factor *Online Learning Engagement*. More specifically, *URL* (β = 0.74, *p* < .001) and *File* (β = 0.60, *p* < .001) accounted for the largest variation, suggesting that accessing and viewing external extra-curriculum materials and course lecture notes are the most salient behaviors for online learning engagement levels. *Assignment* (β = 0.39, *p* < .001) and *Forum* (β = 0.29, *p* < .01) also explained at least 10% of the variance of *Online Learning Engagement*. The SEM model fit statistics and the factor loadings of the measurement model showed that variables extracted from LMS log data can effectively represent students’ levels of online learning engagement. Moreover, behaviors related to accessing intra- and extra-curricular course resources were the most significant indicators of online learning engagement.

## Discussion

Attendance and performance are underexplored in the current online education landscape, although their relationship has been discussed extensively for decades. Even fewer studies have examined how learning technology may mediate this relationship by adopting learning analytics tools to collect objective evidence of in-class attendance and online engagement. When online courses and blended courses are ubiquitous across formal and informal educational contexts, it is especially important to understand the impact of in-class attendance on learning outcome, and how learning technologies could facilitate efficient learning by creating more engagement opportunities and mitigate the absence of in-class interactions. In the relevant studies, self-reported attendance and engagement measured by various instruments still constitute the most common practices. The present study used log data generated from an LMS to explore the relationship between in-class attendance and academic achievement, and further examine how online learning engagement and formative assessment mediate this relationship in the context of a TEL-based course via learning analytics approach and structural equation modeling. We proposed three hypotheses regarding the potential associations among the selected variables. The findings show that all three hypotheses were confirmed. The Baseline model shows that *Attendance* is still significantly correlated with *Course Final Score*, if TEL predictors are excluded. The Mediation model reveals that online learning engagement and formative assessment performance fully mediate the relationship between in-class attendance and academic achievement.

The non-significant, negative direct effect of *Attendance* on *Course Final Score* implies that in-class attendance fails to positively predict performance. The result is contradictory to many previous studies that examined the relationship between attendance and performance in traditional classroom-based courses (Hidayat et al., 2012; Hollett et al., 2020). However, we found consistent results in some of the recent studies conducted in TEL settings (Doggrell, 2020; van Walbeek, 2004). For example, Doggrell (2020) found that there is no direct association between course attendance and academic achievement in medical laboratory science courses when lecture recordings were available. In the 1990s, Durden and Ellis (1995) proposed to use attendance as the proxy of *engagement* and viewed it as one of the most important indicators to performance in addition to student characteristics, such as aptitude and motivation. More recent research studies provided more supporting evidence from various disciplines (Kirby & McElroy, 2003; Louis et al., 2016; Stegers-Jager et al., 2012). However, blended education has gained momentum in recent years and online education has become omnipresent in higher education around the world. The present study suggests that it is no longer valid to simply use attendance to represent motivation or engagement. The SDT argues that higher levels of autonomous forms of motivation rather than external punishment and awards are more effective in promoting students’ engagement and learning (Ryan & Deci, 2020). Previous SDT studies situated in traditional classroom-based courses confirmed the importance of autonomous motivation (Cerasoli & Ford, 2014; Jang et al., 2012; Reeve, 2002, 2013). Results from the current study show that in-class attendance itself does not influence course performance, but that online learning engagement and performance on formative assessments, respectively, fully mediate the relationship between attendance and course performance, after controlling for prior knowledge. Findings of the present study suggest that the new educational technologies reduce the importance of attendance on learning outcomes compared with traditional school contexts. More importantly, educational technologies have been creating more opportunities for engagement.

Nonetheless, we found significant positive mediating effects of both *Online Learning Engagement* and *Performance on Online Formative Assessments* on the relationship between *Attendance* and *Course Final Score*. The results of the bivariate correlations indicate that *Online Learning Engagement* and *Performance on Online Formative Assessments* are two distinct constructs, which can be regarded as two forms of TEL. All the path coefficients on the two mediation pathways are positive, that is, in-class attendance positively impacts the levels of self-regulated online learning and performance on formative assessments, and the two factors subsequently positively influence students’ course final scores. Therefore, students who are more likely to attend the lectures are also more active on learning and self-assessment in LMS. The endogenous nature of attendance is addressed by the mediation effects. The findings reveal similar implications as in Büchele’s (2020) study, where the author used the MSLQ (Pintrich et al., 1991) and the tutorial engagement scale (Handelsman et al., 2005) to evaluate the link between lecture attendance and performance mediated by metacognitive regulation, task value, and tutorial engagement in higher education. Büchele (2020) concluded that it does not matter whether students attend the class with respect to their course success. Rather, levels of cognitive and behavioral engagement mediate the relationship between attendance and performance. In the present study, attendance also does not directly correlate with performance, but has a positive effect on performance through online self-regulated learning and formative assessment. Thus, the two mediation paths further confirm that attendance does not serve as the sole important engagement indicator for predicting academic performance. TEL engagement indicators including self-regulated learning and formative assessment fully mediate the relationship between attendance and performance. Researchers and teachers may re-evaluate the behavioral dimensions when online courses take over in higher education.

In the current climate of online education, where multimedia technologies are widely developed and used, attendance should not be treated as the only proxy for engagement, as it hardly determines academic performance on its own and its impact on learning outcomes can be compensated by various learning technologies. In the present study, we identified two pathways that bridge attendance to performance: one through online engagement and the other through formative assessment. The two engagement indicators incorporate students’ self-regulated learning and assessment in and outside the classroom, which could provide more insights into understanding the interplay of in-class attendance, online learning engagement, and formative assessment in affecting academic performance.

## Educational implications

### Theoretical implications

The present study extends SDT and engagement theory from traditional school-based contexts to a TEL-based course by adding the elements of *Online Learning Engagement* and *Online Formative Assessment* to the behavioral dimension. The mediation effects of the two behavioral engagement indicators on the relationship between attendance and performance are scrutinized to understand how in-class engagement interacts with online engagement and how the two aspects work jointly on performance. Previous studies generally focused only on in-class engagement or only on online engagement to explain individual differences on performance. Findings of the study help fill in the gaps by merging the boundaries of traditional in-class engagement and online engagement to entangle the mechanism of engagement in contemporary education, enhanced by various digital technologies.

### Methodological implications

Methodologically, previous studies on attendance and performance generally used students’ self-recalled information to collect attendance records and adopted self-reported instruments to measure engagement, such as questionnaires or validated tests. However, self-reported measures tend to yield higher measurement errors. This study applied learning analytics methods to analyze the log file data automatically generated in the LMS. Students’ attendance, online learning activities, and performance on formative assessments are well documented in sequences of web-usage log events. Thus, measurement error is greatly reduced. In addition, we performed an SEM analysis, where both observed and latent variables were included, and the interactive effects among variables were estimated simultaneously with minimized error to examine the construct validity of *Online Learning Engagement*.

### Practical implications

In online courses, the classroom is not the only medium that involves engagement. Specifically, online learning platforms are also essential media that deliver course materials and provide digital tools for communication and self-evaluation. Thus, SDT and engagement research should dedicate more attention to engagement in online learning contexts. This study highlights the significance and role of online learning engagement and performance on formative assessments in positively influencing academic performance. Moreover, the SEM results suggest that the availability of diversified digital technologies in education provides more ways to foster learning and engagement in and outside the classroom. We provide instructors with the following instructional suggestions to promote student engagement in blended and online courses. First, it is important to enhance efficient student-faculty interaction and foster positive relationships between students and teachers in technology-enhanced learning (Chiu, 2021a; Paulsen & McCormick, 2020; Robinson & Hullinger, 2008). More specifically, teachers could improve online communication skills so that students could receive high-quality feedback and be emotionally engaged (Chiu, 2021a; Robinson & Hullinger, 2008). Second, instructors are encouraged to design online activities or tasks that facilitate peer discussions and collaborations in class and in digital learning environments (Dumford & Miller, 2018). Peer interactions could connect students online and create opportunities to provide and receive feedback from classmates to improve their learning outcomes. Third, active learning activities are beneficial for facilitating student perceived learning and self-directed learning (Gray & DiLoreto, 2016; Robinson & Hullinger, 2008). Instructors could create a learning community where students can contribute to and be engaged in. In sum, a mixture of educational resources and learning activities is recommended for achieving better course performance in higher education through higher levels of engagement in online environments.

### Limitations

Participants were recruited using a convenience sampling method, so they were all undergraduate students in the Faculty of Education. Further research will be conducted among various participants (i.e., students from different grade levels and programs of study) and in different contexts (i.e., classroom-based courses, online courses, and blended courses) to better understand the impact of attendance and engagement on academic performance in different types of teaching modes. Also, the present study only controlled *Prior Knowledge* as the confounding variable in the two proposed models. Other variables such as gender and affective factors may also potentially influence students’ learning pathways and outcomes. Future studies could collect more demographic information and affective factors to mitigate the extraneous effect of confounding variables.

## Conclusion

This study examines the relationship between attendance and performance mediated by online self-regulated learning and formative assessment in a TEL-based course. The SEM results show that online self-regulated learning and performance formative assessment fully mediate the relationship between attendance and performance. Attendance alone is not a vital determinant of performance, but it positively impacts performance by inducing more active online self-regulated learning and better performance on formative assessments. Findings suggest that mandatory attendance is not a panacea for improving poor academic performance. Instead, more autonomous motivation and engagement are the key to academic success. Further, different forms of educational technologies may mitigate the negative effect of academic absenteeism in school by creating more learning and engagement opportunities online. Therefore, leveraging engagement through self-regulated learning and self-assessment by using a variety of technologies is recommended to promote performance in higher education. Future research should be conducted to extend SDT and engagement theory to TEL education. Specifically, more empirical studies need to be conducted in various domains to further confirm the importance of in-class and online behavioral engagement on performance (Additional file 1).

## Supplementary Information

**Additional file 1.** Supplementary Material.
